# Haplotype of non-synonymous mutations within *IL-23R* is associated with susceptibility to severe malaria anemia in a *P. falciparum* holoendemic transmission area of Kenya

**DOI:** 10.1186/s12879-017-2404-y

**Published:** 2017-04-20

**Authors:** Elly O. Munde, Evans Raballah, Winnie A. Okeyo, John M. Ong’echa, Douglas J. Perkins, Collins Ouma

**Affiliations:** 1 Department of Biomedical Sciences and Technology, School of Public Health and Community Development, Maseno University, Maseno, Kenya; 2 University of New Mexico/KEMRI Laboratories of Parasitic and Viral Diseases, Centre for Global Health Research, Kenya Medical Research Institute, Kisumu, Kenya; 3 Department of Medical Laboratory Science, School of Public Health, Biomedical Sciences and Technology, Masinde Muliro University of Science and Technology, Kakamega, Kenya; 4 Department of Internal Medicine, Centre for Global Health, Health Sciences Centre, University of New Mexico, Albuquerque, NM USA; 5 Centre for Global Health Research, Kenya Medical Research Institute, Kisumu, Kenya; 6 Ideal Research Centre, Kisumu, Kenya

**Keywords:** *Il-23R*, Exon, Genotypes, Haplotypes

## Abstract

**Background:**

Improved understanding of the molecular mechanisms involved in pediatric severe malarial anemia (SMA) pathogenesis is a crucial step in the design of novel therapeutics. Identification of host genetic susceptibility factors in immune regulatory genes offers an important tool for deciphering malaria pathogenesis. The IL-23/IL-17 immune pathway is important for both immunity and erythropoiesis via its effects through IL-23 receptors (IL-23R). However, the impact of IL-23R variants on SMA has not been fully elucidated.

**Methods:**

Since variation within the coding region of *IL-23R* may influence the pathogenesis of SMA, the association between *IL-23R rs*1884444 (G/T), rs7530511 (C/T), and SMA (Hb < 6.0 g/dL) was examined in children (*n* = 369, aged 6–36 months) with *P. falciparum* malaria in a holoendemic *P. falciparum* transmission area.

**Results:**

Logistic regression analysis, controlling for confounding factor of anemia, revealed that individual genotypes of *IL-23R* rs1884444 (G/T) [GT; OR = 1.34, 95% CI = 0.78–2.31, *P =* 0.304 and TT; OR = 2.02, 95% CI = 0.53–7.74, *P =* 0.286] and *IL-23R* rs7530511 (C/T) [CT; OR = 2.6, 95% CI = 0.59–11.86, *P =* 0.202 and TT; OR = 1.66, 95% CI = 0.84–3.27, *P =* 0.142] were not associated with susceptibility to SMA. However, carriage of *IL-23R* rs1884444T/rs7530511T (TT) haplotype, consisting of both mutant alleles, was associated with increased susceptibility to SMA (OR = 1.12, 95% CI = 1.07–4.19, *P =* 0.030).

**Conclusion:**

Results presented here demonstrate that a haplotype of non-synonymous *IL-23R* variants increase susceptibility to SMA in children of a holoendemic *P. falciparum* transmission area.

**Electronic supplementary material:**

The online version of this article (doi:10.1186/s12879-017-2404-y) contains supplementary material, which is available to authorized users.

## Background

One of the most prevalent parasitic infections in humans is *Plasmodium falciparum* malaria [1]. Based on the latest WHO estimates [2], there were 212 million cases of malaria worldwide in 2015 with 90% of the cases occurring in Africa. However, between 2000 and 2015, malaria incidence has declined by 22%. *P. falciparum*-related morbidity and mortality primarily occurs in immune-naïve infants and young children [3, 4]. Severe malaria presents with overlapping clinical sequelae that include severe malarial anemia (SMA), metabolic acidosis, respiratory distress, cerebral malaria (CM) and hypoglycemia [5]. In *P. falciparum* holoendemic transmission areas, such as Siaya County, western Kenya, severe malaria is a predominant cause of under-five morbidity and mortality [6], presenting primarily as SMA (Hb < 6.0 g/dL and any density parasitemia) [7, 8]. The pathophysiology of pediatric SMA is driven by a complex interaction between the human host and *P. falciparum* parasite. The etiological basis of SMA is multifactorial and includes lysis of both infected and uninfected erythrocytes [9–11], an imbalance in production of inflammatory mediators [12–14] that can promote bone marrow suppression and inhibition of erythropoiesis [15–17]. Moreover, we and others have previously demonstrated that SMA is exacerbated by HIV-1 and bacteremia co-infections [18–21].

Interleukin-23 receptor (IL-23R) engagement by IL-23 promotes T-helper 17 (Th17) cell-mediated inflammatory reactions [22, 23]. Th17 cells are pro-inflammatory CD4^+^ effector T-cells that mediate inflammatory process by secreting IL-17 [24]. Binding of IL-23 to IL-23R activates signal transduction via the Janus kinase (JAK)–signal transducer activator of transcription (STAT3/4), and NF-κB pathways [25]. IL-17 is produced by activated T-cells and is involved in priming of T-cells through its ability to stimulate macrophages and some epithelial cells to produce pro-inflammatory mediators [e.g., IL-1, IL-6, TNF-α, NOS-2, metalloproteases known to be important in inflammatory diseases [26, 27]. IL-17 is also important in linking immune responses with erythropoiesis through its ability to enhance proliferation of erythroid precursor cells [28–30]. Although IL-17 has not previously been explored during malaria mono-infection in the cohort of children investigated here, we have shown that IL-17 has a significant positive association with Hb in malaria-infected children with HIV-1 [31]. We have also shown that IL-17 is elevated in children with falciparum malaria and bacteremia co-infections [32], and that IL-23 is elevated in children with SMA [33]. Thus, the IL-23/IL-17 cytokine axis appears to be important in mediating the development of inflammatory reactions in children that develop SMA.

We and others have further shown that in polygenic infectious diseases such as malaria, pathogenesis is influenced by genetic variation in regulatory and/or coding regions of inflammatory mediators and conserved molecular pattern receptors [33–36]. As such, an improved understanding of SMA pathogenesis can be achieved through identification of polymorphisms in genes that mediate the development of severe disease. Although IL-23 receptor variation has not been explored in malaria, carriage of rs10889677CC in the *IL-23R* was associated with increased risk of cancer in Chinese populations [37]. Additional studies have provided evidence on the important role of the IL-23/Th17 axis on immune-mediated diseases such as Crohn’s disease (CD) [38], psoriasis [39, 40] and ankylosing spondylitis (AS) [41]. Collectively, these previous studies established that variations in *IL-23R* influence immune responses and thereby mediates the risk of inflammatory diseases. Even though these diseases present differently from malaria, they all elicit inflammatory reactions in the host. Despite its potential importance, the role of non-synonymous *IL-23R* polymorphisms has not been explored in the context of susceptibility to SMA. To address this gap-in-knowledge, we determined the association between the genotypic and haplotypic structures of non-synonymous *IL-23R* variants (i.e., rs1884444 G/T and rs7530511 C/T) and susceptibility to SMA.

## Methods

### Study site

The study was conducted at Siaya County Referral Hospital (SCRH), western Kenya, a region with holoendemic *P. falciparum* transmission [8]. The region is largely inhabited by the Luo ethnic tribe (~96%), therefore, providing a genetically homogenous population for the study. *Falciparum* malaria prevalence is ~83% in children aged <4 years, with severe disease manifests as SMA [7, 8]. Main mosquito vectors in this area are *Anopheles gambiae s.s, Anopheles arabiensis* and *Anopheles funestus* [42]. The residents receive between 100 and 300 infective mosquito bites per year [43] while intense malaria transmission occurs during April to August and also between November and January seasons [44].

### Study participants

Children [*n* = 369, aged 6–36 months] of both sexes were recruited at SCRH during their first reported hospitalization for treatment of malaria. Recruitment followed a two-tier process of initial screening and enrollment i.e. the parent/guardian of the child received a detailed explanation of the study. Enrollment decisions were made after initial HIV-1 screening of the child and obtaining informed consent from the parent or legal guardian. Written informed consent was administered in the language of choice (i.e., English, Kiswahili or Dholuo). Children with acute malaria were stratified into two categories: non-severe malarial anemia (non-SMA)-defined as a positive smear for asexual *P. falciparum* parasitemia (of any density) and Hb ≥ 6.0 g/dL; and SMA - defined by a positive smear for asexual *P. falciparum* parasitemia (of any density) and Hb < 6.0 g/dL [45]. Children were excluded from the study for any one of the following reasons: children with CM (a rare occurrence in this holoendemic area) and clinical evidence of acute respiratory infection. All children were treated according to the Ministry of Health (MOH)-Kenya guidelines. These included the administration of oral artemether/lumefantrine (Coartem®) for uncomplicated malaria and intravenous quinine for severe malaria, and blood transfusion wherever indicated.

### Sample collection

Venous blood samples (<3.0 mL) were collected in EDTA-containing vacutainer tubes at the time of enrollment, prior to provision of treatment or any supportive care. Blood samples were used for malaria diagnosis, hematological measurements, HIV testing, bacterial culture, and DNA isolation for genetic analyses.

### Laboratory investigations

Hemoglobin levels and complete blood counts (CBC) were performed using the Beckman Coulter ACT diff2™ (Beckman-Counter Corporation, Miami, FL, USA). To determine parasite density, 10% Giemsa-stained thick blood smears were prepared and examined under a compound microscope on high-power magnification. *P. falciparum* parasites per 300 white blood cells (WBC) were determined and parasitemia (/μL) estimated using the total WBC count. To account for the most common causes of severe anemia in the region, anemia-promoting conditions such as HIV-1, bacteremia, sickle-cell trait (HbAS) status, alpha-thalassemia, and glucose-6-phosphate dehydrogenase deficiency (G6PD) were determined and controlled for during analyses. Pre- and post-test HIV counseling was provided for all participants. HIV-1 exposure was determined serologically (i.e., Unigold™ and Determine™), and HIV-1 infection was determined by proviral DNA PCR testing according to our previous methods [20]. Bacteremia was determined using the Wampole Isostat Pediatric 1.5 system (Wampole Laboratories), and blood was processed according to the manufacturer’s instructions. API biochemical galleries (bioMerieux, Inc.) and/or serology were used for identification of blood-borne bacterial isolates. G6PD deficiency was determined by a fluorescent spot test using the manufacturer’s methods (Trinity Biotech Plc., Bray, Ireland), while presence of the sickle cell trait (HbAS) was determined by cellulose acetate electrophoresis as per manufacturer’s conditions (Helena Bio-Sciences, Oxford, United Kingdom).

### DNA isolation for genetic analyses

Blood spots were made on FTA Classic® cards (Whatman Inc., Clifton, NJ, USA), air dried and stored at room temperature until use. DNA was extracted using the Gentra System (Gentra System Inc., Minneapolis, MN, USA) according to the manufacturer’s protocol. The house-keeping gene; human glucose-3-phosphate dehydrogenase (hG3PDH) was used to confirm DNA presence. The genomic DNA (gDNA) was initially amplified using Genomiphi DNA Amplification Kit (Amershan Biosciences SV Corp, CA, USA) according manufacturer’s instructions to obtain more copies.

### *IL-23R* rs1884444 G/T and rs7530511 C/T genotyping

The *IL-23R* rs1884444 G/T, (assay ID: C__11728603_10) and *IL-23R* rs7530511C**/**T (assay ID: C__2990018_10) polymorphisms were genotyped using the TaqMan® 5′ allelic discrimination Assay-By-Design high-throughput method based on the manufacturer’s instructions (Applied Biosystems, Foster City, CA, USA).

### Data analyses

SPSS® statistical software package version 22.0 (IBM SPSS Inc., Chicago, IL, USA) was used for all statistical analyses. Chi-square (χ^2^) analysis was used to examine differences between proportions. Mann-Whitney U test was used for comparisons of demographic and clinical characteristics between the clinical groups. Construction of haplotypes was performed using HPlus software program (Version 2.5). The association between genotypes and/or haplotypes and SMA was determined by logistic regression analysis, controlling for the confounding effects of age, sex, co-infections (HIV-1 and bacteremia), G6PD deficiency, HbAS and alpha-thalassemia status in the regression model at 95% confidence interval (CI) with statistical significance set at *P* ≤ 0.05.

## Results

### Clinical, demographic, and laboratory characteristics of the study participants

The clinical, demographic, and laboratory characteristics of the study participants are presented in Table 1. Parasitemic children (*n* = 369, aged 6–36 months) were categorized as non-SMA (*n* = 207) and SMA (*n* = 162). The distribution of sex was comparable between the clinical groups (*P =* 0.252). Children in the non-SMA group [median (IQR); 13.0 (7.8, 19.0)] were older than children in the SMA group [median (IQR); 9.6 (6.6, 16.3), *P =* 0.019]. Parasitemia levels were comparable between non-SMA [median (IQR); 14,711.7 (3257.4, 48,353.7)] and SMA [median (IQR); 15,656.6 (3258.0, 46,598.1), *P =* 0.760] groups. Axillary temperature at enrollment was also comparable between the groups; non-SMA [median (IQR); 37.6 (36.8, 38.6)]; and SMA [median (IQR); 37.9 (36.9, 38.5), *P =* 0.542], respectively. Children with SMA had an increased respiration rate (breaths/min) [median (IQR); 32.0 (28.0, 40.0)] relative to children with non-SMA [median (IQR); 30.0 (24.0, 40.0), *P =* 0.028]. As expected based on a priori grouping: Hb (g/dL) was lower in SMA [median (IQR); 5.0 (4.3, 5.5)] vs. non-SMA [median (IQR); 9.4 (8.1, 11.2), *P <* 0.001]; hematocrit (%) was lower in SMA [median (IQR); 16.1 (13.5, 17.8)] vs. non-SMA [median (IQR); 29.0 (24.8, 34.6), *P <* 0.001]; and RBC count (×10^12^/μL) was lower in SMA [median (IQR); 2.2 (1.9, 2.7)] vs. non-SMA [median (IQR); 4.2 (3.6, 4.9), *P <* 0.001]. Red cell distribution width (RDW, %) was higher in SMA [median (IQR); 22.9 (21.1, 25.8)] compared to children with non-SMA [median (IQR); 19.6 (16.5, 21.5), *P <* 0.001]. The SMA group also had elevated WBC counts (×10^3^/μL) [median (IQR); 13.0 (9.2, 17.4)] relative to non-SMA group [median (IQR); 10.9 (9.0, 14.3)], *P =* 0.010], while platelet counts (×10^3^/μL) were reduced in children with SMA [median (IQR); 147.5 (105.0, 200.5)] relative to the non-SMA group [median (IQR); 176.0 (122.0, 282.0), *P <* 0.001]. Considering confounding factors demonstrated that bacteremia was more common in children with SMA, 52 (53.1%) relative to non-SMA, 46 (46.9%), *P* = 0.350) while HIV-1 in SMA were 14 (60.9%) compared to non-SMA 9 (39.1%) *P* = 0.090. These distributions were, however, comparable between the two clinical groups. Analysis of the distribution of the genetic factors that have been shown to influence malaria outcome (sickle cell trait, G6PD and alpha thalassemia) also did not reveal any significant differences between the two groups. Sickle cell traits in SMA were 4 (80%) and in non-SMA 1 (20.0%), (*P* = 0.110) while that of G6PD in SMA were 6 (33.3%) and in non-SMA 12 (66.7%) (*P* = 0.310). Even though the proportions of alpha thalassemia were higher in SMA, 41 (53.9%) compared to non-SMA 35 (46.1%), (*P* = 0.070), they were not statistically different (Table 1).

**Table 1:** Demographic, clinical, and laboratory characteristics of the study participants

	Clinical groups^a^		
Characteristics	non-SMA (Hb ≥ 6.0 g/dL) *n* = 207	SMA (Hb < 6.0 g/dL) *n* = 162	*P* value
Sex, *n* (%)			
Male	107 (51.7)	74 (45.7)	0.252^b^
Female	100 (48.3)	88 (54.3)	
Age, (months)	13.0 (7.8, 19.0)	9.6 (6.6, 16.3)	**0.019** ^c^
Parasite/μL	14,711.7 (3257.4, 48,353.7)	15,656.6 (3258.0, 46,598.1)	0.760^c^
Temperature, (°C)	37.6 (36.8, 38.6)	37.9 (36.9, 38.5)	0.542^c^
Respiration rate, (breaths/min)	30.0 (24.0, 40.0)	32.0 (28.0, 40.0)	**<0.028** ^c^
Hematological Parameters			
Hemoglobin, g/dL	9.4 (8.1, 11.2)	5.0 (4.3, 5.5)	**<0.001** ^c^
Hematocrit, %	29.0 (24.8, 34.6)	16.1 (13.5, 17.8)	**<0.001** ^c^
RBC, × (10^12^/μL)	4.2 (3.6, 4.9)	2.2 (1.9, 2.7)	**<0.001** ^c^
RDW, %	19.6 (16.5, 21.5)	22.9 (21.1, 25.8)	**<0.001** ^c^
WBC (×10^3^/uL)	10.9 (9.0, 14.3)	13.0 (9.2, 17.4)	**0.010** ^c^
Platelet Counts (×10^3^/uL)	176.0 (122.0, 282.0)	147.5 (105.0, 200.5)	**<0.001** ^c^
Confounding factors			
Infections			
Bacteremia, *n* (%)	46 (46.9)	52 (53.1)	**0.035**
HIV-1, *n* (%)	9 (39.1)	14 (60.9)	0.090
Genetic factors			
Sickle cell trait	1 (20.0)	4 (80.0)	0.110
G6PD	12 (66.7)	6 (33.3)	0.310
Alpha-thalassemia	35 (46.1)	41 (53.9)	0.070

Data are presented as the median (interquartile range; IQR) and *n* (%) unless stated otherwise

*Abbreviations*: *G6PD* Glucose-6-phospahte dehydrogenase, *RBC* red blood cells, *RDW* red cell distribution width, *WBC* white blood cells

^a^Children with *P. falciparum* malaria (*n* = 369) were categorized as non-SMA (*n* = 207) and SMA (*n* = 162) according to modified definition of SMA (Hb < 6.0 g/dL, with any density parasitemia)

^b^Statistical significance was determined by the Chi-square (χ^2^) analysis

^c^Statistical significance was determined using Mann-Whitney U test

Values in bold are significant *p*-values at a cut-off of *p* < 0.05

### Distribution of *IL-23R* rs1884444 G/T and *IL-23R* rs7530511 C/T genotypes in the clinical groups

Prior to determination of the association between genotypes and SMA, distributions of the *IL-23R* rs1884444G/T and *IL-23R* rs7530511C/T genotypes were determined in the clinical categories. Chi-square (χ^2^) analyses showed that the distribution of the *IL-23R* rs1888444G/T and *IL-23R* rs7530511C/T genotypes were not different between the clinical groups (*P =* 0.278 and *P =* 0.386, respectively, Table 2). *IL-23R* rs1884444G/T genotypes within the non-SMA group were 70.5% GG, 26.6% GT and 2.9% TT, while those in the SMA group were 66.0% GG, 27.8% GT and 6.2% TT (Table 2). Genotypes of *IL-23R* rs1884444G/T in the non-SMA (χ^2^ = 0.087, *P =* 0.767) and SMA (χ^2^ = 2.901, *P =* 0.088) groups were consistent with Hardy-Weinberg Equilibrium (HWE). Allele frequencies of the *IL-23R* rs1884444G/T in the overall study population were 0.82 (G) and 0.18 (T), respectively (Table 2). In addition, the genotypic distribution of the *IL-23R* rs1884444G/T in the overall study population was consistent with HWE (χ^2^ = 2.21, *P =* 0.137).

**Table 2:** Distribution of *IL-23R* 1,884,444 G/T and *IL-23R* rs7530511 C/T genotypes in the clinical groups

	*N* (%) with genotype in group^a^		
Genotypes	Non-SMA (Hb ≥ 6.0 g/dL) (*n* = 207)	SMA (Hb < 6.0 g/dL) (*n* = 162)	*P-value* ^b^
*IL-23R* rs1884444 G/T			
GG, *n* (%)	146 (70.5)	107 (66.0)	
GT, *n* (%)	55 (26.6)	45 (27.8)	0.278^b^
TT, *n* (%)	6 (2.9)	10 (6.2)	
X = 0.18			
*IL-23R* rs7530511 C/T			
CC, *n* (%)	175 (84.5)	128 (79.0)	
CT, *n* (%)	26 (12.6)	28 (17.3)	0.386^b^
TT, *n* (%)	6 (2.9)	6 (3.7)	
X = 0.11			

^a^Data are presented as *n* (%) of children. Children were grouped based on the modified definition of SMA (Hb < 6.0 g/dL, with any density parasitemia)

^b^Statistical significance determined by the χ^2^ analysis. *X*: frequency of the variant allele

The genotypic distribution of the *IL-23R* rs7530511C/T in non-SMA group was 84.5% CC, 12.6% CT, and 2.9% TT, while those in the SMA group were 79.0% CC, 17.3% CT and 3.7% TT (Table 2). In both non-SMA and SMA groups, there was deviation from HWE (χ^2^ = 12.98, *P <* 0.001 and χ^2^ = 6.57, *P =* 0.010), respectively. The major and the minor allele frequency for the *IL-23R* rs7530511C/T in the overall study population was 0.89 (C) and 0.11 (T), respectively (Table 2). In the overall study population, the *IL-23R* rs7530511C/T genotypic distribution showed deviation from HWE (χ^2^ = 18.81, *P <* 0.001).

### Association between *IL-23R* rs1884444 G/T and *IL-23R* rs7530511 C/T genotypes and SMA

The association between individual genotypes of *IL-23R* rs1884444G/T and *IL-23R* rs7530511C/T and susceptibility to SMA was determined using logistic regression analysis, controlling for the confounding effects of age, sex, co-infection (HIV-1 status and bacteremia), HbAS, alpha-thalassemia and G6PD deficiency [46–48]. Relative to the wild-type *IL-23R* rs1884444 (GG), no significant associations with susceptibility to SMA were observed for either the GT (OR = 1.34, 95% CI = 0.78–2.31, *P =* 0.304) or TT (OR = 2.02, 95% CI = 0.53–7.74, *P =* 0.286) genotypes (Table 3). In addition, relative to the wild-type *IL-23R* rs7530511 (CC), neither the CT (OR = 2.60, 95% CI = 0.59–11.86, *P =* 0.202) nor the TT (OR = 1.66, 95% CI = 0.84–3.27, *P =* 0.142) were associated with susceptibility to SMA. Moreover, to provide a more global representation, we also included analysis based on the WHO cut-off of SMA (SMA; Hb < 5.0 g/dL and any density parasitemia). We however did not observe any significant association between the *IL-23R* rs1884444G/T and *IL-23R* rs7530511C/T genotypes and SMA in this study population (Table 3).

**Table 3:** Association between *IL-23R* rs1884444 G/T and *IL-23R* rs7530511 C/T genotypes and SMA

	SMA (Hb < 6.0 g/dL			SMA (Hb < 5.0 g/dL		
Genotypes	OR	95% Cl	*P* value	OR	95% CI	*P* value
*IL-23R* rs1884444 G/T				*IL-23R* rs1884444 G/T		
GG, (*n* = 253)	Ref	-	-	Ref	-	-
GT, (*n* = 100)	1.34	0.78–2.31	0.304	3.69	0.89–5.13	0.174
TT, (*n* = 16)	2.02	0.53–7.74	0.286	1.39	0.73–2.64	0.316
*IL-23R* rs7530511 C/T				*IL-23R* rs7530511 C/T		
CC, (*n* = 303)	Ref	-	-	Ref	-	-
CT, (*n* = 54)	2.60	0.59–11.86	0.202	2.02	0.43–9.42	0.372
TT, (*n* = 12)	1.66	0.84–3.27	0.142	2.49	0.76–5.37	0.102

Children (*n* = 369) with *P. falciparum* malaria were categorized on the basis of presence or absence of severe malarial anemia SMA (defined as Hb < 6.0 g/dL, with any density parasitemia). Odds ratios (OR) and 95% confidence intervals (CI) were determined using logistic regression, controlling for age, sex, co-infections (HIV-1 status and bacteremia), sickle cell trait (HbAS), G6PD deficiency, and alpha-thalassemia. The reference groups in the logistic regression analysis were the homozygous wild-type genotypes

### Association between *IL-23R* rs1884444 G/T and *IL-23R* rs7530511 C/T haplotypes and SMA

Using logistic regression analysis models, controlling for the confounding effects of age, sex, co-infection (HIV-1 status and bacteremia), HbAS, alpha-thalassemia, and G6PD deficiency [8, 46–49], we determined the association between carriage of the *IL-23R* rs1884444 and rs7530511 haplotype constructs and SMA. These analyses revealed that there was no association between carriage vs. non-carriage of the *IL-23R* rs1884444G/rs7530511C (GC) haplotype and SMA (OR = 0.49, 95% CI = 0.18–1.33, *P =* 0.161, Table 4). Susceptibility to SMA was also not influenced by carriage vs. non-carriage of either the GT (OR = 1.04, 95% CI = 0.33–3.31, *P =* 0.949) or TC (OR = 0.97, 95% CI = 0.52–1.81, *P =* 0.923) haplotypes (Table 4). However, carriage of the TT haplotype was associated with a significant increase in susceptibility to SMA (OR = 1.12, 95% CI = 1.07–4.19, *P =* 0.030, Table 4). Likewise, using the WHO cut-off for SMA, only the TT haplotype was associated with the risk of SMA (OR = 2.50, 95% CI = 1.18–5.29, *P* = 0.016, Table 4).

**Table 4:** Association between *IL-23R* rs1884444 G/T and *IL-23R* rs7530511 C/T

	SMA (Hb < 6.0 g/dL)			SMA (Hb < 5.0 g/dL)		
*IL-23R haplotypes*	OR	95% CI	*P*-value	OR	95% CI	*P*-value
GC, (*n* = 342)	0.49	0.18–1.33	0.161	0.34	0.13–1.17	0.107
GT, (*n* = 17)	1.04	0.33–3.31	0.949	1.92	0.52–7.06	0.328
TC, (*n* = 67)	0.97	0.52–1.81	0.923	1.02	0.49–2.15	0.955
TT, (*n* = 56)	1.12	1.07–4.19	**0.030**	2.50	1.19–5.29	**0.016**

Haplotypes and SMA

Children with *P. falciparum* malaria (*n* = 369) were grouped based on the modified definition of SMA (i.e., Hb < 6.0 g/dL, with any density parasitemia). Odds ratios (OR) and 95% confidence intervals (CI) were determined using logistic regression model controlling for age, sex, co-infections (HIV-1 and bacteremia) sickle cell trait (HbAS), G6PD deficiency, and alpha-thalassemia. The reference groups in the regression analysis were non-carriers of the respective haplotypes

Values in bold are significant *p*-values at a cut-off of *p* < 0.05

## Discussion

In *P. falciparum* holoendemic transmission areas, one of the most common clinical outcomes of malaria is SMA. To further provide additional information on genes that condition susceptibility to SMA, we investigated the role of the IL-23/IL-17 cytokine pathway by determining the genetic association between non-synonymous mutations of *IL-23R* rs188444G/T and rs7560511C/T polymorphisms and susceptibility to SMA. The study showed that individual genotypes in *IL-23R* (rs188444G/T and rs7530511C/T) were not independently associated with susceptibility to SMA. However, carriage of the *IL-23R* rs188444T/rs7560511T (TT) haplotype was associated with increased susceptibility to SMA [using both modified (Hb < 6.0 g/dL) and WHO (Hb < 5.0 g/dL) definition of SMA].

The protein encoded by the *IL-23R* gene located on chromosome 1 is a subunit of the receptor for IL-23 sub-unit alpha (IL-23A) which pairs with the receptor molecule IL-12β1, both of which are required for IL-23A signaling [50]. The IL-23R/IL-12β1 dimer binds to IL-23, which is made up of a p19 protein and IL-12p40 sub-units [51]. In addition to its expression on memory T-cells, IL-23R is present on other immune cells, including activated antigen presenting cells (APCs), natural killer cells, and monocytes, all of which are involved in host-defense against invading pathogens [52–54]. Genetic variation in IL-23R plays an important role in determining the efficacy of cellular immune responses [55]. The current study demonstrates that genotypic variants of *IL-23R* [i.e., rs1884444 (G/T) and rs7530511 (C/T)] are not individually associated with susceptibility to SMA.

The *IL-23R* (rs188444 G/T) is located at codon 3 in exon 2 of the *IL-23R* and results in a histidine-to-glutamine substitution. This G to T change is known to be responsible for changes in the signal peptide of the *IL-23R* and results in exon skipping, alternative splicing, or malformation [55] resulting in alteration of the receptor-ligand binding specificity. Previous studies showed that *IL-23R* rs1884444 variation is associated with susceptibility to esophageal and gastric cancer, schistosomiasis-associated immune reconstitution inflammatory syndrome, and inflammatory bowel disease [56–58]. However, consistent with a study in Chinese adults with pulmonary tuberculosis (PTB) and drug-resistant PTB [59], we found no association in the current investigation between rs1884444 variants and malaria disease outcomes.

The *IL-23R* rs7530511 C/T polymorphism results in a replacement of proline for leucine at codon 310 (P310L). The rs7530511 C/T is located adjacent to the motif sequence, WQPWS, present in the membrane-transmembrane proximal *IL-23R* domain, and is capable of altering receptor affinity [60], a variation that can influence differential production of downstream molecules. Individual genotypes of rs7530511 in our population, however, did not show any independent associations with susceptibility to SMA, despite earlier observations that the rare TT genotype of the rs7530511 was associated with autoimmune conditions, such as Graves’ disease (GD) [60]. This could be explained by the autoimmune nature of GD which is thyroid gland specific while *P. falciparum* malaria that affects multiple organs.

Considering the fact that haplotypes within particular genes are often capable of exposing genetic combinations which can moderate or interact to produce effects that are not observable with individual genotypes [33, 61], we therefore constructed haplotypes of *IL-23R* rs1884444 G/T and rs7530511 (C/T). The current study revealed that the carriage of *IL-23R* rs1884444/rs7530511 (TT) haplotype was associated with an increased risk of SMA. This observation implies that carriage of both mutant genotypes (TT) is an important genetic risk factor for developing SMA once a child becomes infected with *P. falciparum*. Although presently undetermined, one can speculate that the TT haplotype may amplify exon skipping and/or mRNA splicing, resulting in altered affinity of the receptor for IL-23 (ligand) binding [55, 60]. Since IL-23/T-helper 17 axis leads to the production of IL-17, and other pro-inflammatory mediators, the TT haplotype may potentially inhibit the generation of pro-inflammatory mediators that aid in controlling malarial infections. However, the complex interplay between successfully controlling an infection and the generation of inflammatory-derived anemia during a malaria infection is difficult to discern at the molecular level [62]. IL-17 is a prototypical example of such complexity since IL-17 bridges immune and hematopoietic regulation by stimulating early stage erythroid progenitors (i.e., burst forming unit erythroid, BFU-E) [63], and in the opposite context, inhibits late stage erythroid progenitors [30, 64, 65]. Further studies are required to delineate the influence of genetic polymorphisms within *IL-23R* on differential expression and production of inflammatory mediators to unravel the molecular mechanisms through which the IL-23/T-helper 17 axis collectively influences the development of malarial disease outcomes.

## Conclusion

In summary, the current study provides evidence that haplotypes of the *IL-23R* alter the risk of developing SMA in pediatric populations. We present data on a homogenous population consisting of ~96% Luo ethic group, a key characteristic in genetic studies of this nature. Furthermore, our study underscores the use of haplotype analysis approach in genetic association studies as it presents unique association not identifiable using individual allele or genotype analysis. We are currently investigating the impact of multi-haplotypes of *IL-23R* variants on longitudinal outcomes of malaria including mortality. In addition, due to the fact that polymorphisms may have distinct effects in different ethnic groups presenting with varied forms of severe malaria, studies across various ethnic groups are warranted to decipher fully the impact of *IL-23R* variation on susceptibility to severe malaria anemia.

## Additional file

Additional file 1: These are details of the raw data for the study participants (*N* = 369) used in the analyses of results presented in the current paper. (XLS 384 kb)

## Abbreviations

APCs: Antigen presenting cells
BFU-E: Burst forming unit erythroid
CM: Cerebral malaria
G6PD: Glucose-6-phosphate dehydrogenase
Hb: Hemoglobin
HbAS: Hemoglobin AS type
HDP: High density parasitemia
HWE: Hardy Weinberg Equilibrium
IL-23R: Interleukin −23 receptor
IQR: Interquartile range
MOH: Ministry of Health
NF-κB: Nuclear factor kappa-light-chain-enhancer of B cells
SMA: Severe malarial anemia
SNP: Single nucleotide polymorphisms
WBCs: White blood cells
